# Sub-high Temperature and High Light Intensity Induced Irreversible Inhibition on Photosynthesis System of Tomato Plant (*Solanum lycopersicum* L.)

**DOI:** 10.3389/fpls.2017.00365

**Published:** 2017-03-16

**Authors:** Tao Lu, Zhaojuan Meng, Guoxian Zhang, Mingfang Qi, Zhouping Sun, Yufeng Liu, Tianlai Li

**Affiliations:** ^1^College of Horticulture, Shenyang Agricultural UniversityShenyang, China; ^2^Key Laboratory of Protected Horticulture of Education Ministry and Liaoning ProvinceShenyang, China; ^3^Collaborative Innovation Center of Protected Vegetable Surrounds Bohai Gulf RegionShenyang, China

**Keywords:** photosynthesis, photoinhibition, antioxidant system, PSII, PSI

## Abstract

High temperature and high light intensity is a common environment posing a great risk to organisms. This study aimed to elucidate the effects of sub-high temperature and high light intensity stress (HH, 35°C, 1000 μmol⋅m^-2^⋅s^-1^) and recovery on the photosynthetic mechanism, photoinhibiton of photosystem II (PSII) and photosystem I (PSI), and reactive oxygen (ROS) metabolism of tomato seedlings. The results showed that with prolonged stress time, net photosynthetic rate (Pn), Rubisco activity, maximal photochemistry efficiency (Fv/Fm), efficient quantum yield and electron transport of PSII [Y(II) and ETR(II)] and PSI [Y(I) and ETR(I)] decreased significantly whereas yield of non-regulated and regulated energy dissipation of PSII [Y(NO) and Y(NPQ)] increased sharply. The donor side limitation of PSI [Y(ND)] increased but the acceptor side limitation of PSI [Y(NA)] decreased. Content of malondialdehyde (MDA) and hydrogen peroxide (H_2_O_2_) were increased while activity of superoxide dismutase (SOD) and peroxidase (POD) were significantly inhibited compared with control. HH exposure affected photosynthetic carbon assimilation, multiple sites in PSII and PSI, ROS accumulation and elimination of *Solanum lycopersicum* L.

## Introduction

“Liaoyuanduoli” is an infinite-growth-typed tomato plant containing lots of properties including high quality and diseases resistance, high and stable yield, low temperature and low light tolerance, extensive adaptability and so on. In addition, it is suitable for cultivation in both open field and protected field and it has a large area of cultivation in north China. However, it always faces high temperature and high light intensity stresses hindering its growth and development in long season cultivation over summer. The optimum growth temperature and irradiance condition of tomato is 15–32°C and 500–800 μmol⋅m^-2^⋅s^-1^, and, 35°C is defined as its sub-high daytime temperature (Zhang et al., 2005). Previous studies have demonstrated that the yield and quality decline of tomato under sub-high temperature was related to the affection of plant photosynthesis (Zhang et al., 2008; Yuan et al., 2010a). Different studies with controversy showed that the reason of net photosynthetic rate (Pn) decline can be non-stomatal factor (by reducing leaf stomatal conductance with CO_2_ supply disruption) (Ristic et al., 2009) or stomatal factor (by increasing the gas diffusion impedance, CO_2_ solubility, Rubisco affinity to CO_2_ in mesophyll cells) (Ainsworth and Rogers, 2007; Hozain et al., 2010). Consequently, conducting a systematic study of the effects of sub-high temperature and high light intensity on tomato leaf photosynthetic apparatus and the plant’s recovery capability is important.

Photoinhibition mentions the decrease in photosynthetic efficiency under the circumstance in which the input of photons goes beyond the demand for photosynthesis. Photosystem II (PSII) has long been viewed as the most sensitive component to high temperature and high light (Critchley and Smillie, 1981; Ragni et al., 2008; Huang et al., 2016) including photooxidation and photoinhibition of PSII in plants. Among more than 25 kinds of subunits in PSII protein complex, D1 protein is located in the PSII reaction center and is encoded by the chloroplast-localized *psbA* gene. Furthermore, D1 protein is the major target site damaged by various environmental stresses (Michoux et al., 2016). A process termed D1 turnover would happen to counter the impairment and to keep photosynthetic activity when D1 protein was damaged. Under high light intensities, when the capability for repair does not match up with D1 degradation rate, photodamage cumulates, aggravating photoinhibition (Aro et al., 1993; Yamamoto et al., 2013). Recent studies have recommended that high temperature did not make grave damage to PSII; alternatively, it suppressed its repair mechanism (Tyystjärvi, 2012). Photosystem I (PSI) is seldom damaged and is impaired generally in chilling-sensitive plants when suffering cold stress (Ivanov et al., 1998; Li and Zhang, 2015). However, recent studies illustrate that PSI gets damaged as electron flow from PSII exceeds its electron acceptors’ capacity to deal with, when occurring, the impairment is practically irreversible (Scheller and Haldrup, 2005). In addition, high temperature or high light will also break down PSI (Barth et al., 2001; Huang et al., 2015). Given this, it is necessary to find out the mechanisms underlying the inhibition of PSI and PSII involved in sub-high temperature and high light intensity.

Under normal condition, the excited energy is transmitted to PSII and PSI reaction centers where charge separation happens and photosynthetic electron transportation is initiated by singlet excited state of chlorophyll. If photons energy is inordinate and excited chlorophyll is not able to drive photochemistry, it then results in photoinhibition and brings out reactive oxygen species (ROS) via the Mehler reaction in the chloroplasts, like singlet oxygen (^1^O_2_), superoxide (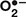), hydroxyl radical (•OH) and hydrogen peroxide (H_2_O_2_) (Suzuki et al., 2012). ROS accumulation is highly destructive to photosynthetic system and carbohydrate metabolism. Additionally, the accumulation of ROS has been regarded as one of the fast kinetic events exposing plants to diverse stresses. Plants have got expeditious antioxidant systems for ROS removal (Yuan et al., 2010b), including non-enzymatic (such as ascorbate, glutathione, and carotenoids) and enzymatic [e.g., superoxide dismutase (SOD), peroxidase (POD), catalase (CAT), ascorbate peroxidase (APX), glutathione reductase (GR), dehydroascorbic reductase (DHAR) and monodehydroascorbate reductase (MDHAR)]. Generation and scavenging system of ROS have attracted more attention due to their different roles in the defense of plants against heat, chilling, high light and so on (Ragni et al., 2008; Wang et al., 2009; Huang et al., 2016). Upon the equilibrium is disrupted under adverse conditions, photooxidation, photobleaching and even cell death occurs. Photooxidation results in irreversible inactivation of the photosynthetic system and retardation of the recovery, which leads to the overall degradation of PSII and sever diminishing of plant productivity (Hihara and Sonoike, 2013). In earlier studies, ROS directly break down PSII on thylakoid membranes or PSII complexes (Nishiyama et al., 2011). Recent studies have illustrated that ROS performs mainly by suppressing the repair of damaged PSII (Nishiyama and Murata, 2014). As heat-induced photoinhibition in plants is closely relevant to the antioxidant system, the mechanisms of “how does the photodamage happened and what’s the role of ROS in the related ‘processes”’ need to be clarified.

Changes in gas exchange, activity of Rubisco, photochemical activity of PSII and PSI, generation and removal of ROS, the expressions of related genes were analyzed with tomato seedlings under sub-high temperature and high light intensity at 35°C and 1000 μmolm^-2^s^-1^ (HH). Their recovery at 25°C and 500 μmolm^-2^s^-1^ was also determined. The results provide insights into the relationship among CO_2_ assimilation, photosynthetic electron transport, photoinhibition, and ROS metabolism of tomato leaves under HH stress.

## Materials and Methods

### Plant Materials and Temperature and Irradiance Treatments

‘Liaoyuanduoli’ of tomato (*Solanum lycopersicum* L.) were germinated and cultivated in 12 cm × 12 cm nutrition pots within a greenhouse (average day/night temperatures, 25°C /15°C) with natural light at a relative humidity of 60%.

Tomato seedlings were separated into four growth rooms (QIUSHI ENVIRONMENT, Zhejiang, China) at the six-leaf stage from the greenhouse, with a total of 40 plants per room. Four groups were subjected to different temperature (25°C, 35°C/15°C) or irradiance (500, 1000 μmol⋅m^-2^⋅s^-1^) conditions (**Table 1**). In each growth room, the light source was metal halide lamps (HQI, 400 W, Osram, Munich, Germany). After 5 days of treatment, the plants were moved to the control room. All parts were permitted to recover for 10 days and all treatments were initiated at day 0.

**Table 1:** The four temperature and irradiance combinations in phytotrons.

Treatments	Temperature of daytime (°C)		Illumination intensity (μmol⋅m^-2^⋅s^-1^)	
	25	35	500	1000
CK	6:00–18:00		6:00–18:00	
HT	6:00–8:0016:00–18:00	8:00–16:00	6:00–18:00	
HL	6:00–18:00		6:00–11:0013:00–18:00	11:00–13:00
HH	6:00–8:0016:00–18:00	8:00–16:00	6:00–11:0013:00–18:00	11:00–13:00

Before measurement, plants were dark-adapted for 20 min. All measurements such as gas exchange and chlorophyll fluorescence parameters were performed on the fourth fully expanded functional leaves adopting at least three seedlings from each treatment. Sampling for biochemical and physiological analyses was carried out on 0, 1, 3, 5 days after the treatments and on 5 and 10 days for the recovery. Subsequently, the samples were frozen in liquid nitrogen and stored at -80°C.

### Measurement of Gas Exchange Parameters

As described by Yamori et al. (2011), gas exchange, chlorophyll fluorescence, and P_700_ redox state were simultaneously obtained by DUAL-PAM-100 and GFS-3000 measuring systems (Heinz Walz, Effeltrich, Germany).

The net Pn, transpiration rate (Tr), stomatal conductance (Gs), and vapor pressure deficit (VPD) were recorded with constant irradiation (500 μmol⋅m^-2^⋅s^-1^, PAR). Stomatal limitation value (Ls) was determined based on a computational formula 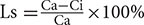 (Zhang et al., 2005), where Ca and Ci is atmospheric and intercellular CO_2_ concentration respectively. The observation data were recorded after leaf state stabilization.

### Measurement of Chlorophyll Fluorescence and P_700_ Parameters

Chlorophyll fluorescence and P_700_ parameters were measured from the same leaves that were previously used for photosynthetic measurement. The dark-adapted and light-adapted maximal fluorescence (Fm and Fm’) were obtained at 20 kHz with a 1 s pulse of 6000 μmol⋅m^-2^⋅s^-1^ of “white light.” The dark-adapted and light-adapted initial fluorescence (Fo and Fo’) were measured by switching on the modulated irradiation of less than 0.1 μmol⋅m^-2^⋅s^-1^ PPFD on the leaf surface. With a saturation pulse’s help, P_700_ red was determined in a given state. Pm and Pm’ are analogous to Fm and Fm’ respectively, they were given by the same means as the former fluorescence parameters by applying a saturation pulse after pre-illumination with far-red light (Linkosalo et al., 2014). Likewise, the formulas of other fluorescence parameters are presented in **Table 2**. All the leaves used for measurement were dark-adapted for 20 min earlier.

**Table 2:** Chlorophyll fluorescence and P_700_ parameters, descriptions, and the calculation equations.

Parameter	Description	Formula
Fv/Fm	The maximal photochemistry efficiency of PSII	Fv/Fm = (Fm-Fo)/Fm
Fv’/Fm’	The efficiency of excitation energy capture by open PSII reaction centers	Fv’/Fm’ = (Fm’-F_0_’)/Fm’
Fv/Fo	The potential activities of PSII	Fv/Fo = (Fm-Fo)/Fo
*qP*	The photochemical quenching coefficient	*q*P = (Fm’-Fs)/(Fm’-F_0_’)
NPQ	The non-photochemical quenching coefficient	NPQ = (Fm-Fm’)/Fm’
Y(II)	The efficient quantum yield of PSII	Y(II) = (Fm’-Fs)/Fm’
Y(NO)	The yield of non-regulated energy dissipation of PSII	Y(NO) = Fs/Fm
Y(NPQ)	The yield of regulated energy dissipation of PSII	Y(NPQ) = 1-Y(II)-Y(NO)
Y(I)	The effective quantum yield of PSI	Y(I) = 1-Y(ND)-Y(NA)
Y(ND)	The donor side limitation of PSI	Y(ND) = 1-P700_red_
Y(NA)	The acceptor side limitation of PSI	Y(NA) = (Pm-Pm’)/Pm
ETR	The relative electron transport rate	ETR = Yield × PAR × 0.5 × 0.84

### Measurement of Rubisco Activity

Leaf sample was homogenized with cooled extraction buffer (1 mM EDTA, 50 mM pH 7.5 Tris-HCI, 10 mM MgCl_2,_ 10% PVP, 12.5% glycerin, and 10 mM β-mercaptoethanol) in a pre-chilled mortar, and then centrifuged at 15,000 ×*g* for 15 min at 4°C (Liu Y.F. et al., 2012). The activity of Rubisco was measured by enzyme linked immunosorbent assay-sandwich technique with a Rubisco ELISA KIT (Yanyu, Shanghai, China) following the instruction.

### Identification of Lipid Peroxidation and Membrane Damage

Because malonaldehyde (MDA) is the end product of antioxidant enzyme activities and lipid peroxidation, its accumulation is used as an indicator of lipid peroxidation (Sudhakar et al., 2001). The amount of MDA was determined by the thiobarbituric acid test and H_2_O_2_ determination was did by estimating the titanium hydro-peroxide complex as described previously (Spicher et al., 2016).

Besides, free proline estimation was performed and improved according to sulfosalicylic acid method and soluble protein was measured on the basis of Gong et al.’s (2013) description.

To detect the integrity and stability of membrane lipids, the values of the electrolyte leakage are usually used to evaluate the plant’s ability of tolerate stress (Chen et al., 2004). Relative electrolyte conductivity K was calculated by 
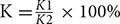
. Leaf sample was shaken in 5 mL double distilled water at room temperature for 2 h to test conductivity, recorded as K_1_, and then boiled for 30 min to test conductivity, saved as K_2_. Cell damage degree α was calculated by 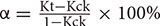, K_t_ and Kck represented relative electrical conductivity of treated and control leaf separately.

### The Histochemical Staining and *In situ* Location of 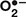 and H_2_O_2_

Histochemical staining for 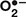 and H_2_O_2_ followed the method of Wang and Zeng (1997) with NBT and DAB respectively.

*In situ* localization of 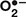 was performed using Dihydroethidium (DHE) according to Liu F. et al. (2012). In the case of H_2_O_2_, the highly sensitive, cell-permeable probe 2′,7′-dichlorofluorescin diacetate (DCFH-DA) was utilized based on Capone et al. (2013). Images were captured with an inverted fluorescence microscope system (Axio Observer A1, Zeiss, Germany) using standard filters and collection modalities for DHE yellow fluorescence (excitation 515 nm; emission 525 nm) and for DCFH-DA green fluorescence (excitation 488 nm; emission 525 nm).

### Antioxidant Enzyme Activities

Leaf sample was homogenized with cold 25 mM HEPES buffer (pH 7.8, 2% PVP and 0.2 mM EDTA), and then centrifuged at 12,000 ×*g* for 20 min at 4°C. The supernatant was used for enzyme analysis.

The activity of total superoxide dismutase (T-SOD) was determined by measuring its ability to inhibit the photochemical reduction of NBT at 560 nm. CAT and POD activities were determined by absorbance at 240 and 470 nm due to the decrease in extinction of H_2_O_2_ and the decrease of oxidized phenols of H_2_O_2_ (Wei et al., 2011) respectively.

### Protein Extraction, Electrophoresis and Western Blot Analysis

Immunoblot assays of total protein extracts using anti-D1 antibodies were performed to evaluate overall D1 level which is in parallel with PSII activity. Total proteins were extracted according to Michoux et al. (2016) with some modifies. Tomato sample was homogenized with cooled protein extraction buffer (50 mM pH 7.5 HEPES, 330 mM d-Sorbitol, 2 mM Na_2_EDTA and 5 mM MgCl_2_), and then centrifuged at 4°C with 3,000 ×*g* for 5 min. Subsequently, re-homogenized the sediment was in the same protein extraction buffer. Determined total chlorophyll concentration spectroscopically after extraction with 80% (v/v) buffered acetone using the formula C (mg.L^-1^) = 7.12 A_660_ + 16.8 A_642.5_. Then, the membrane suspension containing 100 μg chlorophyll was centrifuged at 3,000 ×*g* for 5 min. To obtain a final chlorophyll concentration of 0.5 mg.L^-1^, the sediment was re-homogenized in protein sample buffer (5 mM pH 6.8 Tris-HCl, 5% Glycerin, 2% SDS, 0.05% Bromophenol blue and100 mM DTT).

Protein samples were incubated for 5 min at 95°C prior to gel loading and separated by SDS-PAGE at 220V using 15% resolving gels with 4% stacking gels containing 10% SDS. After electrophoresis, the proteins were then transferred to PVDF membranes (Millipore, Molsheim, France) and blocked with 5% non-fat milk. The membrane was subsequently incubated with antibodies raised against D1 (Agrisera).

Immunoblots were probed using a Western Blotting Detection Kit (Tiangen, Beijing, China) and the Western Blot Imaging System (Azure Biosystems c600, America). Finally, D1 protein was quantified using the Imagelab System (Bio-Rad, America).

### Total RNA Extractions and RT-qPCR Analysis

Total RNA extraction was done with RNA prep pure plant total RNA extraction kit (Tiangen, Beijing, China) following the manufacturer instruction. Then, reversely transcribed RNA samples into cDNAs as described Jain and Khurana (2009).

Real-time PCR analysis was performed using SYBR^@^ *Premix Ex Taq*^TM^ (TaKaRa, Dalian, China), the ABI 7500 Real Time PCR system and Software 7500 V 2.0.6 (Applied Biosystems, USA) with three replications according to the manufacturer instructions. Each pair of primer was designed using Primer Express 5.0 (Applied Biosystems, USA). The primer sequences are listed in **Table 3**.

**Table 3:** Response gene accession numbers and primer sequences described in this study.

Category	Accessing NO.	Primer sequences (5’-3’)
*rbcL*	L14403.1	**F**5′-GCTGCCGAATCTTCTACTGG-3′
		**R**5′-TTTTCTCCAACAACGCGCTC-3′
*rbcS*	M15236.1	**F**5′-TGTGGAAGTTGCCTATGTTTGG-3′
		**R**5′-GCACTTGACGCACATTGTCG-3′
*_(Cu/Zn)_SOD*	AF034411.1	**F**5′-ACCAGCACTACCAATTCTTTCT-3′
		**R**5′-GGGGTTTAGGGGTAGTGACA-3′
*_(Mn)_SOD*	M37151.1	**F**5′-GGCACCTACCTCTTCACTCA-3′
		**R**5′-GGATTGTAATGTGGTCCTGTTGA-3′
*APX*	AF413573.1	**F**5′-ATGACGCGGGGACTTACAA-3′
		**R**5′-GGCTGGAGAAGTTTCAGTGC-3′
*GR*	NM001247314.2	**F**5′-GGCTACATCTTTGAGCTCACC-3′
		**R**5′-CGGAGAGGCTTGATAGGGTT-3′
*PsbA*	AY568719.1	**F**5′-TGCTCATAACTTCCCTCTAGACC-3′
		**R**5′-AGCACCCTCTTGACAGAACA-3′
*PsbB*	XM010321037.1	**F**5′-GGGCATATATGATACCTGGGC-3′
		**R**5′-ACAATCCAGCCTTCTCCTCC-3′
*PsbC*	DQ347959.1	**F**5′-GATCCCGGCCGTTTACTTTC-3′
		**R**5′-ACAGGGTCAGAGGGATCAAAA-3′
*PsbD*	AM087200.3	**F**5′-GTGTATTGGGCGCTGCTTT-3′
		**R**5′-TCTTCGGCTTGAGTTGGGTT-3′
*PsbP*	NM001247180.3	**F**5′-TCATGCACTAAGATCTAGCCCT-3′
		**R**5′-GCGTTGCTGGCATCATCTT-3′
*PsbQ*	NM001247180.3	**F**5′-TGTCTTGGATGGTAGCCTCC-3′
		**R**5′-CTGTTGGGCTCTGACAGTGA-3′
*PsbR*	NM001247113.3	**F**5′-TGGCAAGCACAGTAATGAGC-3′
		**R**5′-TCTCAAGGCCATGCTTCCAT-3′
*PsbS*	U04336.1	**F**5′-TGTTCCTACCTTCTCTTCCTTTG-3′
		**R**5′-ATTGAAACAGAGCGAGAGAGT-3′
*PsbX*	X63007.1	**F**5′-GCCCTGCTAGAACATCCTCT-3′
		**R**5′-GAACCAATGGCAGCAGTACC-3′
*Ftsh6*	NM001247262.1	**F**5′-GCAAATCCCAAGACTTCTCCA-3′
		**R**5′-CCACTAGTACTCAACAGCTTCC-3′
*Ftsh-Like*	AY277738.1	**F**5′-AAGATAGAGGAATCAGCAAAGGT-3′
		**R**5′-TCCAGCTCAGATTTTGCTTCA-3′
*PsaA*	J03558.1	**F**5′-TGTTTGCCCCTCTTTCCTCT-3′
		**R**5′-GGCATCCAATCAGCTGACAT-3′
*PsaB*	DQ347959.1	**F**5′-GCTGCATTATATACCCACCACC-3′
		**R**5′-TCTTCATTTTGCTCCGGATTGT-3′
*PsaD*	M21344.1	**F**5′-TCAAGCTTCCCTCTTCACCC-3′
		**R**5′-GGGTTACTGAGACGGTGGAT-3′
*PsaA(A1Like)*	AM087200.3	**F**5′-CTTTGGCGAGCATCTGGAAT-3′
		**R**5′-CAAGCCAATTTTAGCGCTGC-3′
*PsaB(A2Like)*	DQ347959.1	**F**5′-CTGTTTCACGTAGCTTGGCA-3′
		**R**5′-GAGTAAAAGCTTCCACGGCC-3′
*Actin*	Q96483	**F**5′-TGTCCCTATTTACGAGGGTTATGC-3′
		**R**5′-AGTTAA ATCACGACCAGCAAGAT-3′

### Data Processing and Analysis

The data were analyzed using SPSS 20 Software (IBM SPSS STATISTICS, USA). The figures were prepared by Origin 9.0 Software (OriginLab, Northampton, MA, USA). Values presented are means ± one standard deviation (SD) of three replicates. Duncan’s multiple range test (DMRT) was utilized to compare significant differences between treatments.

## Results

### Effects on Photosynthetic Parameters and Rubisco Activities

#### Effects on Photosynthesis

**FIGURE 1:**
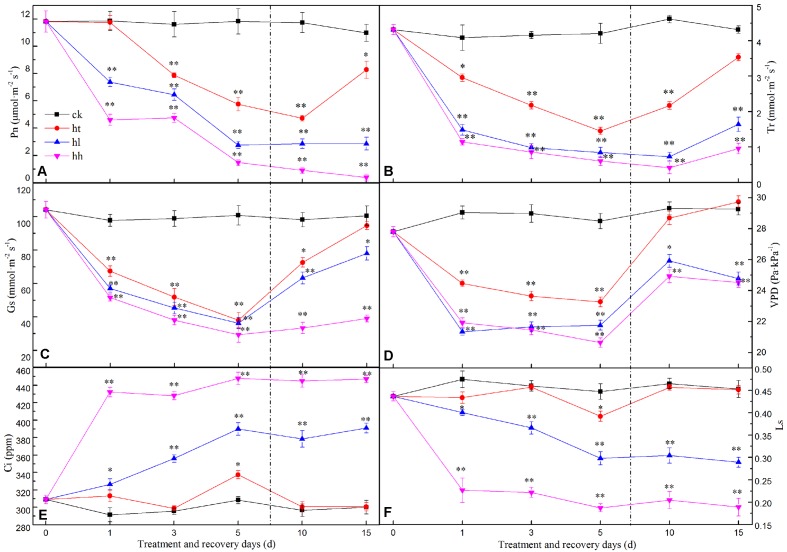
**Effects of sub-high temperature and high light treatment and recovery on the net photosynthetic rate** (Pn, **A**), transpiration rate (Tr, **B**), stomatal conductance (Gs, **C**), vapor pressure deficit (VPD, **D**), intercellular CO_2_ concentration (Ci, **E**), and stomatal limitation value (Ls, **F**) of tomato leaves. The vertical dashed line indicates the transfer of plants back to the control phytotron. Data are the means of six replicates with standard errors shown by vertical bars. ^∗^indicates significant difference (*P* ≤ 0.05), and ^∗∗^indicates a highly significant difference (*P* ≤ 0.01).

In this study, photosynthetic performance was significantly inhibited after the plants were exposed to sub-high temperature or high light intensity stress for 5 days (**Figure 1**). Compared with control, Pn, Gs and Ls decreased faster and reached a significantly lower value after HL and HH treatment (**Figures 1A,C,F**), but, Ci increased markedly (**Figure 1E**). HT treatment declined Pn slower than HL and HH treatment, in addition, the values of Ls and Ci were nearly to the control levels. The adverse stresses not only suppressed photosynthetic capacity but also seriously inhibited transpiration, for instance, all treatments caused a large and fast decrease of Tr and VPD (**Figures 1B,D**).

#### Effects on Rubisco Enzyme

**FIGURE 2:**
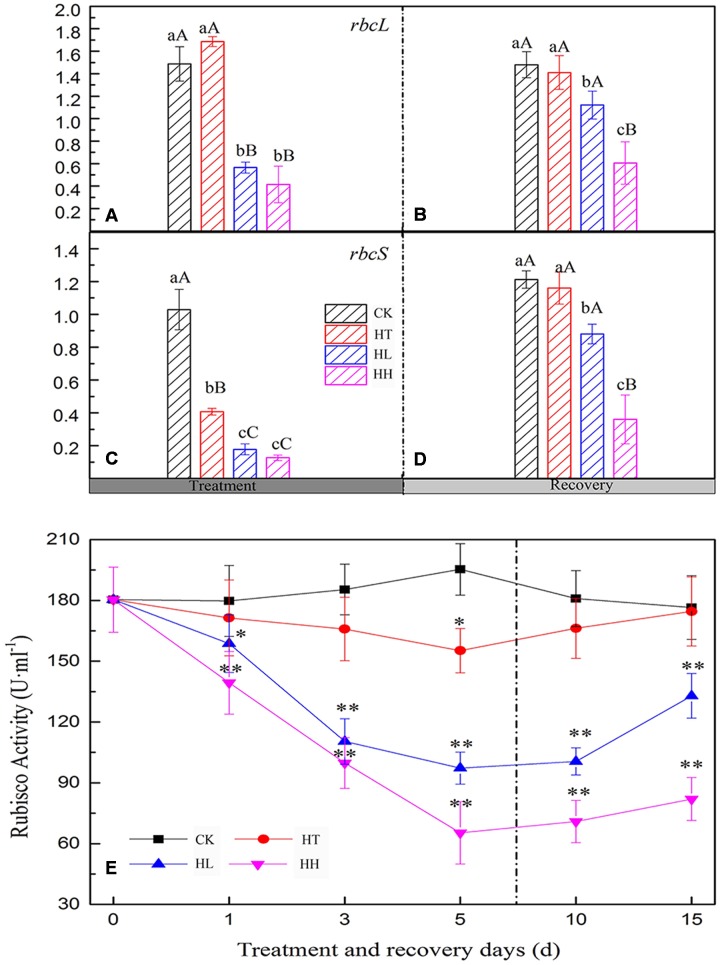
**Effects of sub-high temperature and high light treatment and recovery on the expression of large (*rbcL*) and small (*rbcS*) subunits of Rubisco, and Rubisco activities of tomato leaves**. Lower-case letters and capital letters represent significant difference levels based on One-way ANOVA (*P* = 0.05 and *P* = 0.01) respectively. Mean ± *SE*, *n* = 3.

Compared to the control, plants treated with HL and HH, the activity of Rubisco was highly inhibited, and with prolonged stress, it was reduced aggravatingly (**Figure 2E**). However, HT negatively affected Rubisco activity slightly.

HL and HH treatment significantly reduced expressions of *rbcL* and *rbcS* with respect to the control (**Figures 2A,C**). Expressions of *rbcL* and *rbcS* decreased by 58 and 81% under HL in comparison with the control. HH further decreased that by 71 and 87% respectively. Likely, expression of *rbcS* under HT exposure decreased significantly, but there is no significant difference with control of *rbcL* expression.

#### Photosynthesis Recovery

Photosynthesis recovery processed pretty lento. That is, 75.3, 26.1, and 3.5% recovery rates of Pn inhibition were obtained from HT, HL and HH for 10 days separately, with similar recovery trends for Tr, Gs, VPD, Ci and Ls at moderate temperature and suitable light intensity (**Figures 1A–F**). The Rubisco activity of HL increased significantly during the recovery period for 10 days, and a recovery rate of 75% was obtained. More seriously, Rubisco activity of HH increased very slowly, and less than 42% recovery rate was obtained, which is significantly lower than control. The same enzyme of HT was almost close to the control plants (**Figure 2E**). Additionally, the expressions of *rbcL* and *rbcS* recovered to the control levels except HH-treated plants (**Figures 2B,D**).

### Effects on Photochemical Activity and Encoding Gene Expression of PSII

#### Effects on PSII Photochemical Activity

**FIGURE 3:**
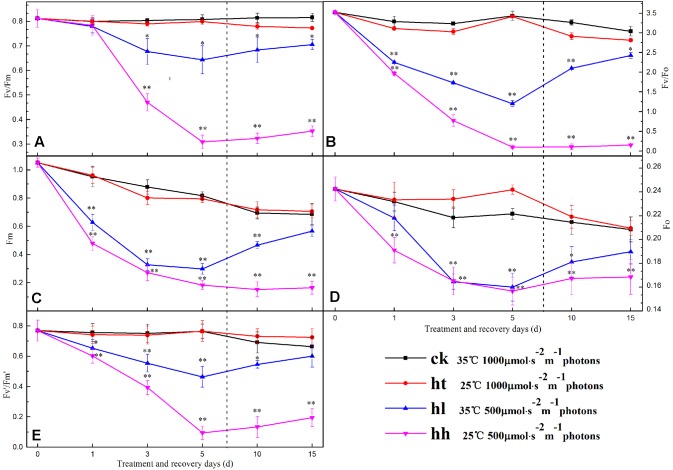
**Effects of sub-high temperature and high light treatment and recovery on the maximal photochemical efficiency of PSII** (Fv/Fm, **A**), potential activities of PSII (Fv/Fo, **B**), the maximal fluorescence (Fm, **C**), the initial fluorescence (Fo, **D**), and efficiency of excitation capture by open PSII reaction centers (Fv’/Fm’, **E**) of tomato leaves.

**FIGURE 4:**
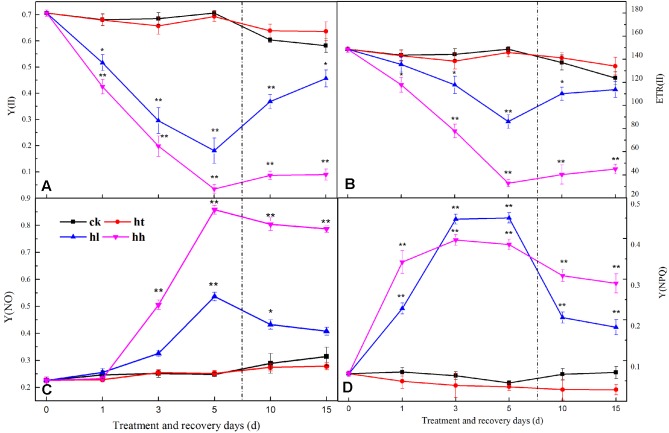
**Effects of sub-high temperature and high light treatment and recovery on Y(II), efficient quantum yield of PSII (A)**; ETR(II), relative electron transport rate of PSII **(B)**; Y(NO), yield of non-regulated energy dissipation of PSII **(C)**; Y(NPQ), yield of regulated energy dissipation of PSII **(D)** of tomato leaves.

In comparison with the control, the ratio of Fv/Fm, Fv/Fo, Fv’/Fm’ and the value of Fm and Fo were significantly lower with HL and HH treatment for 5 days (**Figure 3**). Meanwhile, sub-high temperature single stress affected the above parameters slightly and there is no significant difference between HT and CK samples. Photochemical quenching (*qP*) also showed a large decrease once the plants were exposed to high light intensity (such as HL and HH), and, non-photochemical quenching (NPQ) showed opposite trend (**Table 4**). Besides, Y(II) significantly decreased with prolonged HL and HH stress, associated with significantly increase of Y(NO) and Y(NPQ) (**Figure 4**). At HH-treated plants for 5 days, Y(II) decreased by 92% whereas Y(NO) and Y(NPQ) increased by 71 and 86% respectively compared to the control. Both ETR(II) of HL and HH were significantly decreased (**Figure 4B**). Compare to the control, ETR(II) decreased by 43% and 74% at HL and HH respectively.

**Table 4:** Effects of sub-high temperature and high light treatment and recovery on photochemical quenching coefficient (*qP* and qL), and non-photochemical quenching coefficient (qN and NPQ) of tomato leaves.

Time(d)	Treatments	*qP*	qN	qL	NPQ
5	HH	0.26 ± 0.25^bB^	0.16 ± 0.06^aA^	0.24 ± 0.23^bB^	0.97 ± 0.05^aA^
	HL	0.40 ± 0.06^bB^	0.69 ± 0.04^bB^	0.26 ± 0.06^bB^	0.86 ± 0.26^bB^
	HT	0.91 ± 0.02^aA^	0.21 ± 0.02^aA^	0.69 ± 0.04^aA^	0.52 ± 0.03^aA^
	CK	0.92 ± 0.01^aA^	0.18 ± 0.02^aA^	0.74 ± 0.02^aA^	0.38 ± 0.02^aA^
15	HH Recovery	0.25 ± 0.04^bB^	0.18 ± 0.04^bB^	0.25 ± 0.03^bB^	0.70 ± 0.05^bA^
	HL Recovery	0.90 ± 0.01^aA^	0.27 ± 0.04^bA^	0.71 ± 0.02^aA^	0.59 ± 0.05^aA^
	HT Recovery	0.88 ± 0.03^aA^	0.28 ± 0.08^bA^	0.67 ± 0.05^aA^	0.55 ± 0.11^aA^
	CK Recovery	0.84 ± 0.06^aA^	0.40 ± 0.08^aA^	0.67 ± 0.07^aA^	0.49 ± 0.14^aA^

*Lower-case letters and capital letters represent significant difference levels based on One-way ANOVA (*P* = 0.05 and *P* = 0.01) respectively*.

#### Effects on Gene Expression and D1 Protein Turnover of PSII

**FIGURE 5:**
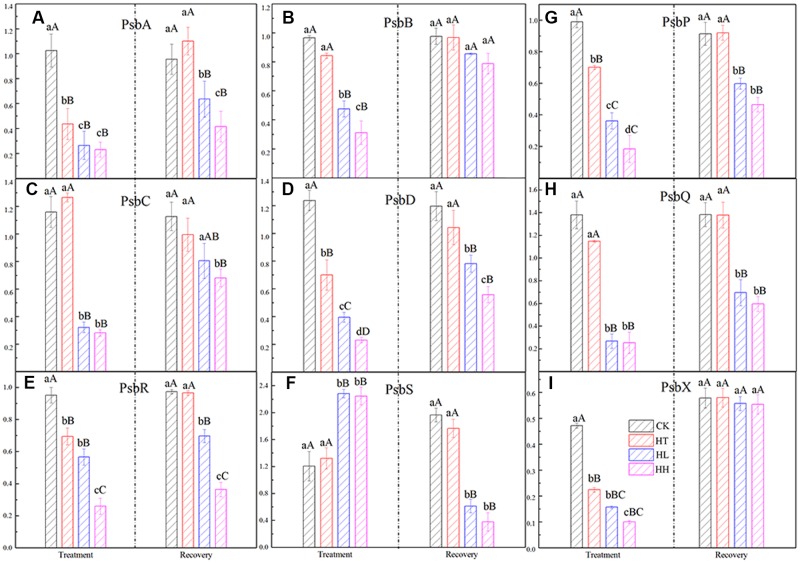
**Effects of sub-high temperature and high light treatment and recovery on expression of related coding genes of the PSII reaction center complex of tomato leaves**.

Compared to the control, expression of genes coding PSII reaction center (such as *PsbA, PsbB, PsbC, PsbD* and so on) were significantly declined by HL and HH treatment (**Figure 5**). Among that, there is significant difference of gene expression (e.g., *PsbB, PsbD, PsbR* and *PsbP*) between plants treated with HL and HH (**Figures 5B,D,E,G**). In addition, only the expression of *PsbS* showed a large increase trend (**Figure 5F**), it was nearly twofold expression values compare to the control.

**FIGURE 6:**
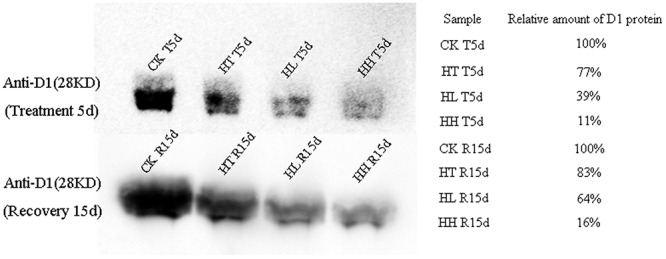
**Effects of sub-high temperature and high light treatment and recovery on the amount of D1 protein in thylakoids of tomato leaves**. Amount of D1 were determined by immunoblotting with specific D1-N terminal antibodies (28 kD). Relative densitometry values for D1 are presented on the right. CK T5d and CK R15d samples represent 100% respectively.

At the protein expression level, D1 protein content was evidently inhibited by sub-high temperature or high light intensity for 5 days (**Figure 6**). The gray-scale value declined by 23, 61, and 89% of HT, HL and HH samples with respect to the control samples.

#### Recovery of PSII

In the present study, relative fluorescence parameters of PSII reaction center decreased in varying degrees. After plants were transferred to moderate temperature and suitable light intensity condition for 5–10 days, average recovery rates were 79–90% of HL-treated plants and below 45% of HH-treated plants (**Figures 3**, **4**, and **Table 4**). While the recovery of PSII from HT inhibition occurred more quickly and mostly reached that of the control level.

For D1 protein turnover in this investigation, once HL or HH was removed, the recovery of transcription and translation of D1 protein were 73%, 64% and 43%, 16% to the control level respectively (**Figures 5A**, **6**). And, the difference is significant in comparison with control.

### Effects on Photochemical Activity and Gene Expression of PSII

#### Effects on PSII Photochemical Activity

**FIGURE 7:**
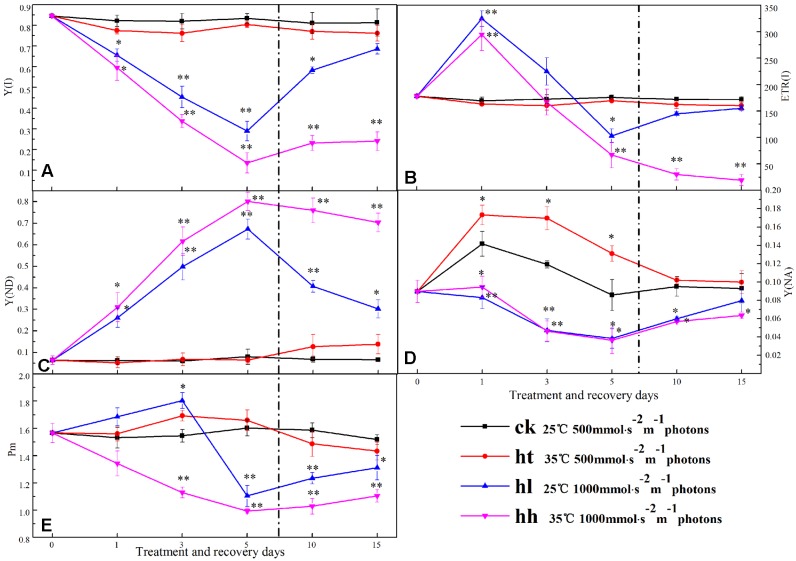
**Effects of sub-high temperature and high light treatment and recovery on Y(I), the effective quantum yield of PSI (A)**; ETR(I), relative electron transport rate of PSI **(B)**; Y(ND), donor side limitation of PSI **(C)**; Y(NA), acceptor side limitation of PSI **(D)**; Pm, the maximum change of P_700_ signal **(E)** of tomato leaves.

Energy conversion of PSI was significantly influenced after 5 days of HL and HH treatment (**Figure 7**). In comparison with that of control, Y(I) of HL and HH treatment groups were significantly lower accompanied with the large increase of Y(ND) (**Figures 7A,C**). Y(I) of HL and HH declined by 80 and 64%, while Y(ND) increased almost six-fold and eight-fold compared with the control. At the begin of treatment, ETR(I) was increased by HL and HH, then declined rapidly. For 5 days, 43 and 57% reduction rate were obtained from HL and HH treatment samples (**Figure 7B**). The value of Y(NA) also changed significantly at HL and HH conditions compared with the control (**Figure 7D**). Data of that showed a similarly low value.

#### Effects on PSII Gene expression

**FIGURE 8:**
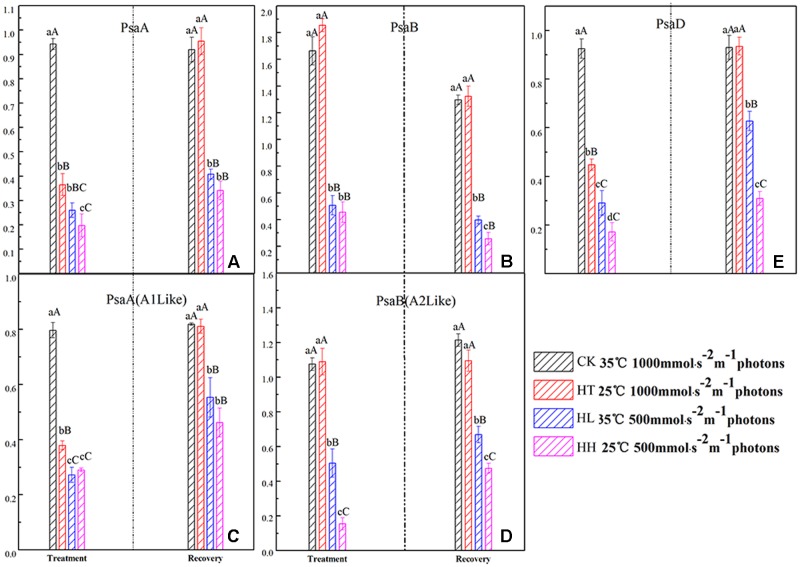
**Effects of sub-high temperature and high light treatment and recovery on expression of related coding genes of the PSI reaction center complex of tomato leaves**.

In the present study, the expressions of *PsaA* and *PsaB* coding core proteins of PSI were seriously decreased by HL and HH (**Figure 8**), and the amount of gene expression was significantly lower than control. HT slightly affected the expression of *PsaB* but also inhibited the expressions of *PsaA* and *PsaD* compared with control.

#### Recovery of PSII

Once stress conditions were removed, complete recovery of Y(I) and ETR(I) was observed in plants of HT and HL (**Figure 7**). However, Y(I) and ETR(I) of HH were still significantly lower than the control accompanied by high Y(ND) value. The same recovery tendency was also found in A1 and A2 protein transcription of PSI (**Figure 8**).

### Effects on Activated Oxygen Metabolism

#### ROS Generation, Lipid Peroxidation and Thylakoid Membrane Damage

**FIGURE 9:**
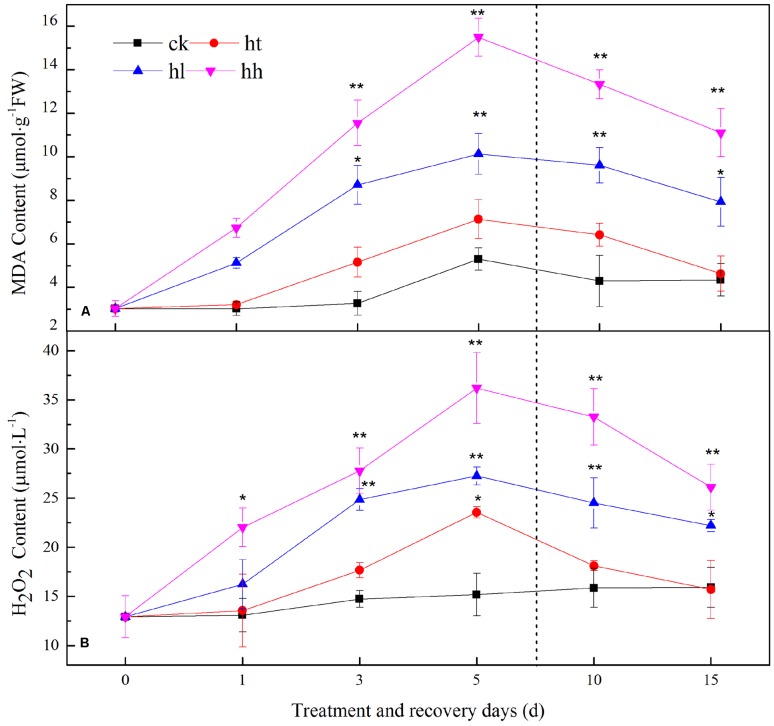
**Effects of sub-high temperature and high light treatment and recovery on lipid peroxidation (expressed as MDA content, A**) and free radical species (mainly H_2_O_2_, **B**) of tomato leaves.

In our study, fluorescent labeled observations for H_2_O_2_ and 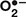 detection in guard cells with cell-permeable probe DCFH-DA and DHE was aligned well with histochemical observations for that detection of leaves with DAB and NBT staining (**Supplementary Figure S2**). Furthermore, MDA and H_2_O_2_ progressively increased under HL (increased by 90 and 83%) and HH (increased by 2 and 1.5 times) stress (**Figure 9**). An extremely significant difference was found between these two plant groups with the control. What’s more, the ROS burst significantly decreased the content of soluble protein and free profine, and seriously impaired the cell membrane integrity and (**Supplementary Figure S3**). HT slightly increased the content of MDA and H_2_O_2_, accompanied by relative electrical conductivity, but there is no significant difference compared with control.

#### Effects on ROS Scavenging Metabolism and Relative Gene Expression

**FIGURE 10:**
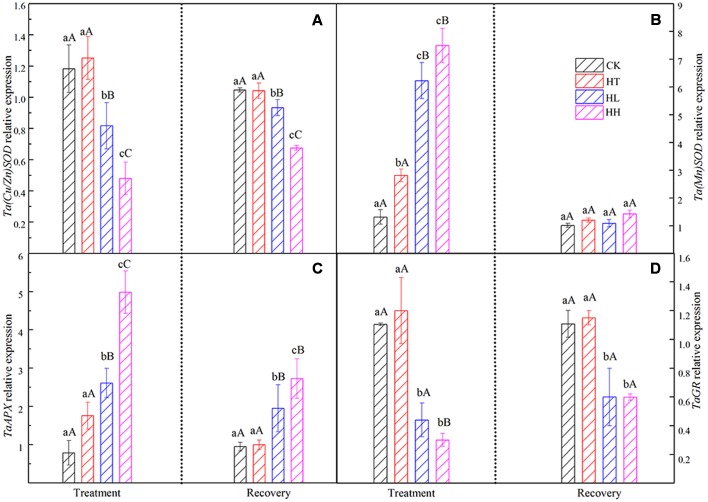
**Effects of sub-high temperature and high light treatment and recovery on genes expression of antioxidant enzymes of tomato leaves**.

Plants own ROS scavenging enzymes including SOD, POD, CAT, APX and GR. In this experiment, HT increased the above enzymes’ activities slightly. However, HL and HH inhibited the activity of SOD and POD and nudged up the activity of CAT (**Table 5**). At the same time, gene expression of APX was significantly higher than the control and expression of GR showed the opposite trend under HL and HH (**Figure 10**).

**Table 5:** Effects of sub-high temperature and high light treatment and recovery on the activities of antioxidant enzymes (SOD, POD and CAT) of tomato leaves.

Time(d)	Treatments	T-SOD (U.g^-1^FW)	SOD (U.g^-1^FW)	POD (U.g^-1^FW.min^-1^)	CAT (U.g^-1^FW.min^-1^)
5	HH	24.30 ± 1.59^cC^	22.03 ± 2.68^cC^	29.28 ± 3.67^bB^	15.48 ± 2.97^bB^
	HL	37.18 ± 1.44^bB^	31.00 ± 1.84^bB^	39.37 ± 0.67^bB^	20.37 ± 1.29^bB^
	HT	44.53 ± 3.09^aA^	44.03 ± 3.45^aA^	65.34 ± 3.17^aA^	7.67 ± 0.34^aA^
	CK	47.05 ± 1.71^aA^	43.34 ± 1.91^aA^	64.75 ± 3.49^aA^	6.03 ± 0.26^aA^
15	HH Recovery	46.71 ± 2.09^aA^	44.75 ± 0.75^aA^	25.17 ± 4.41^cB^	12.67 ± 0.81^bA^
	HL Recovery	46.95 ± 2.20^aA^	43.95 ± 0.29^aA^	48.67 ± 2.28^bA^	15.67 ± 1.70^aA^
	HT Recovery	47.67 ± 2.43^aA^	43.90 ± 1.08^aA^	60.37 ± 2.61^aA^	19.06 ± 0.30^aA^
	CK Recovery	46.27 ± 1.47^aA^	43.75 ± 2.60^aA^	66.25 ± 4.62^aA^	18.09 ± 1.39^aA^

*Lower-case letters and capital letters represent significant difference levels based on One-way ANOVA (*P* = 0.05 and *P* = 0.01) respectively*.

#### Recovery of ROS Metabolism

The levels of MDA and H_2_O_2_ returned to the control values when plants subjected to HT stress were transferred to the suitable environment for 10 days. But HH and HL-treated plants showed 25–52% and 38–75% recovery rate respectively (**Figure 9**). During the recovery period, POD and CAT activities in HH could not recover, they were still significantly lower than control (**Table 5**). Besides, *Ta(Cu/Zn)SOD, TaAPX and TaGR* relative expression levels were below the control values (**Figure 10**).

## Discussion

Under natural conditions plants are exposed to combination of multiple stress factors but the studies on sub-high temperature and high light intensity stresses of plants are rather rare. In the present study, effects of HT, HL and HH on photosynthetic metabolism, energy conversion and electron transport of PSII and PSI, lipid peroxidation and ROS metabolism in *Solanum lycopersicum* L. were analyzed. Abiotic stress strongly affected plant growth and development. In our study, HT made the plant stems thicken, HL led to leaf etiolation, HH caused serious growth and development obstacle as shown in **Supplementary Figure S1**. Besides, the content of Chl a and Chl b, ratio of Chl a and Chb were both decreased by HT, HL and HH (Supplementary Table S1).

The decline of Pn accompanied by the decrease of Gs, Tr, Ls and the increase of Ci (**Figure 1**) indicated that non-stomatal limitation was the main reason for HL and HH-induced decrease of plant photosynthetic capacity (Gerganova et al., 2016). However, as Ci and Ls of HT were almost similar to control level, the decline of Pn was caused by mixture of stomatal and non-stomatal limitation (Tu et al., 2015). It is well known that Rubisco is a key restriction enzyme regulating photosynthetic carbon assimilation in the Calvin cycle (Yamori et al., 2012). The activity and expression state of Rubisco differs in leaves depending upon environmental conditions (Helbling et al., 2011). In this study, HT promoted the transcription of the large (*rbcL*) subunit of Rubisco (**Figure 2A**) accompanied by the substantial reduction in photosynthetic capacity (**Figure 1A**) and slight inhibition of Rubisco activity (**Figure 2E**). Some previous studies conducted under moderate heat stress reported similar results (Simkin et al., 2003). However, HL and HH significantly decreased Rubisco activity as well as the transcription of both *rbcL* and *rbcS* (**Figure 2**), with prolonged stress, the difference became greater. In other words, HL and HH down regulated transcription of the catalytic unit and activity unit of Rubisco, causing the inhibition of Rubisco activity to assimilate carbon and the reduction of photosynthetic capacity (Salvucci, 2008). In addition, it was proposed that the key photosynthetic enzyme activity of RuBPcase was susceptible to sub-high temperature or high light, especially the negative impact of the latter.

The large decrease of Pn followed by a significant and reversible decline of Fv/Fm also ruled out that as the primary target site of HL and HH, PSII inhibition happened particularly in the recovery process (**Figure 3A**). This result is in agreement with previous discoveries (Ragni et al., 2008; Huang et al., 2010). Besides, reduced photosynthesis can lead to the accumulation of excessive photon energy and photoinhibition of PSII. In botany’s living molecule, Fv/Fo reflecting the potential PSII activity was suppressed more seriously by HL and HH than Fv/Fm (**Figure 3B**), which was also demonstrated in maize and bitter gourd (Wang H.Y. et al., 2003; Zhu et al., 2011), implying that a portion of the PSII reaction center was impaired. Inactivation of PSII reaction center is divided into two kinds of types that are reversible inactivation and destruction of reaction center according to Yamamoto et al. (1998). In this study, Fo of HT was higher than control and went back to the control level in the recovery process (**Figure 3D**). That is to say, short-term sub-high temperature induced reversible inactivation of PSII reaction center on the basis of our processing material, which is consistent with the results of previous studies (Chen et al., 2004). While the large decline in Fo and Fm indicated that HH caused destruction of PSII reaction center (**Figures 3C,D**). Whereas, HL induced parts of reversible inactivation (Fo can be retrieved) and irreversible inactivation (Fo has not fully recovered) (Li et al., 2009, 2011). Genty et al. (1989) proposed a model of PSII function and distinguished between the *qP* and Fv’/Fm’ of these centers. The former is used to measure PSII redox state and the latter is a measure of the efficiency of antenna conversion and the supply of energy that reaches the PSII reaction centers. The decline of PSII photochemical efficiency is due to *qP* and Fv’/Fm’. In the present study, it’s distinctly that the decrease of Fv/Fm and Fv/Fo was accompanied by a similar decline of both Fv’/Fm’ and *qP* under HL and HH (**Figure 3E**, **Table 4**). This finding confirmed that although a rapid down regulation of PSII photochemical activity played an essential photoprotection role under HL and HH in response to the photosynthetic carbon metabolism’s inhibition, the changes in PSII reaction center could not surmount these adversities stresses. Therefore, the inactivation of PSII under HL and HH is not only a result but the cause of the photosynthetic capacity loss. As a consequence, the absorbed energy may become excessive due to lower energy conversion efficiency, photochemical efficiency of PSII and lower energy requirement for carbon fixation. At the same time, the noteworthy increase of NPQ indicated that excessive energy was chiefly dissipated as heat and that PSII photoprotective process occurred (**Table 4**). These data gave support to the idea that the regulation of PSII reaction center by the non-photochemical quenching of excitation energy can be operated to a considerable degree to reduce the excitation energy reaching the PSII reaction centers in our research. A reduction of CO_2_ assimilation capability in the Calvin cycle decreased the NADPH and ATP requirement leading to decreased electron transport to tally with the lower demanding for NADPH and ATP.

HL and HH stress induced a significant decrease of ETR(II) (**Figure 4B**), which was ascribed to both the decline in the number of open PSII reaction centers and the efficiency of energy capture by these open centers. This discovery confirmed that linear electron passage through PSII was brought down. Besides, our results revealed that Y(II) and Y(I) of HL and HH were significantly lower than the control. This may be due to electron transport inhibition between PSII and PSI (**Figures 4A**, **7A**). According to Zhang et al. (2014), the blocking of electron transport was mainly caused by the decrease of PQ pools, which might lead to the phosphorylation of thylakoid protein, increasing of cyclic electron flow, alleviation of ATP deficit, and increment of proton motive force, thereby down regulating the PSII antenna via the qE mechanism (Yi et al., 2005).

Y(NPQ) and Y(NO) are two important indicators of photoprotection and photodamage respectively. In the present study, the significant increase of Y(NO) accompanied by a significant increment of Y(NPQ) under HL and HH (**Figures 4C,D**). The increase of Y(NO) indicated that the PSII super-complex had been impaired and D1 turnover had been disturbed by excess light energy (**Figures 5**, **6**). While, higher Y(NPQ) implied that there was still photochemical energy conversion (such as NPQ mechanisms) or protective regulatory mechanisms (such as heat dissipation) to dissipate the light energy absorbed by plants.

As one of the super-complexes of photosystems, PSII holds an intriguingly large number of low molecular weight proteins. In higher plants, the core subunits of PSII is four large transmembrane proteins: D1, D2, CP43 and CP47 which are coded by *PsbA*, *PsbB*, *PsbC* and *PsbD* separately. And, oxygen-evolving complex (OEC) is coded by *PsbQ* and *PsbP.* In addition, *PsbS* which cords photoprotective protein is important in qE mechanism, *PsbR* is responsible for the protein assembly and *PsbX* is important for accumulation of functional PSII (Garcia-Cerdan et al., 2009; Wittenberg et al., 2014). In our study, HL and HH-induced significantly decline of the above genes expression except *PsbS* (**Figure 5**). We suggested that HL and HH caused a down regulation transcription level of D1 protein, the inner peripheral proteins and OEC proteins, accompanying with a significant down regulation of the assembled and functional proteins’ expression. As a result, the accumulation of excitation pressure lead to photooxidative damage to PSII reaction center and the degradation of the D1 protein. Meanwhile, D1 protein content was evidently inhibited by environmental stresses (**Figure 6**). Our observation proved the consistency between translation and transcription level of D1 protein under HL or HH. By comparison, HT mainly induced down regulation transcription of *PsbA, PsbP, PsbR* and *PsbX* (**Figures 5A,E,G,I**), there by inhibited activity of PSII and oxygen release.

Few researches on PSI have studied isolated thylakoid membranes using artificial electron acceptors or donors (Liu Y.F. et al., 2012). In this experiment, a Dual-PAM-100 fluorometer was used to assess the PSI of tomato plants *in vivo*. In comparison with control, lack of Y(ND) change under HT stress indicated that PSI was not inhibited by HT (**Figure 3C**). Nevertheless, the decline of Y(I) in HL and HH-treated tomato leaves resulted from the decrease of the acceptor side limitation of PSI [as reflected by Y(NA)] and the increase of the donor side limitation of PSI [as reflected by Y(ND)] (**Figures 7C,D**). These discoveries suggested that the reduced proportion of electron carriers on the acceptor side of PSI and excessive amount of light energy to PSI at the donor side can be both used as an indicator of PSI photoinhibition (Liu Y.F. et al., 2012). Additionally, this phenomenon may be caused by two reasons: firstly, photodamage of PSII or Cytb_6_/f caused by excessive energy leading to the blocking of electron transfer and accumulation of electron in the PSI donor side, resulting photoinhibition damage to PSI (**Figures 3**, **4**, and **Table 4**). Secondly, electron accumulation in the PSI acceptor side that was caused by the disordered Calvin cycle due to the inactivation of Rubisco for assimilation (**Figure 2**). We speculated that photooxidation caused the photoinhibition of PSI.

Photosystem I complex is consisted by two large subunits and a plurality of small subunits. *PsaA* and *PsaB* encode A1 and A2 protein respectively, *PsaD* is connected to Fd which is the PSI acceptor and is responsible for the stabilizing of PSI (Croce and Bassi, 1998). Light energy is accepted and transferred to P_700_ which is located at the inside of the thylakoid membrane of PSI reaction center by photosynthetic pigments of PSI. Once energy in the reaction center is not utilized or dissipated, it would lead to the accumulation of ROS and damage to the PSI complex. In the present study, the transcription levels of A1 and A2 protein were significantly down regulated by HL and HH (**Figure 8**). We inferred that, under the condition of our processing, excessive amount of excitation energy in PSI led to irreversible photodamage of PSI which was caused by photooxidation.

Plants use O_2_ as a terminal electron acceptor in both Mehler reaction and photorespiration to preserve the chloroplasts. However, under environmental stress conditions, extra electrons consolidate with O_2_ resulting in the production of ROS such as 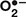 and H_2_O_2_ (Jaspers and Kangasjarvi, 2010). In our study, the ROS burst seriously impaired the membrane system pursuant to MDA values, and cell membrane integrity (**Figure 9A**, **Supplementary Figure S3**). The accumulation of ROS led to membrane peroxidation within the thylakoid and chloroplasts. ROS production is not a necessary symptom of cellular dysfunction, but might symbolize a necessary signal to adjust the cellular machinery to vary circumstances (Jaspers and Kangasjarvi, 2010; Suzuki et al., 2012).

Plants own ROS scavenging enzymes including SOD, POD, CAT, APX and GR. In this experiment, HT increased the above enzymes’ activities in consistent with a previous study findings (Li et al., 2011). HL and HH inhibited the activity of SOD and POD but nudged up the activity of CAT (**Table 5**). At the same time, expression of APX was significantly higher than the control and that of GR showed the opposite trend (**Figure 10**) under HL and HH. These findings are deemed to be a response to the augmented generation of ROS and one of the protective mechanisms against oxidative stress. They do activate protective mechanisms so that plants can live with diverse stresses (Scebba et al., 2001). In short, the accumulation of ROS is very detrimental to the photosynthetic systems, and, the lower electron transportation activity between PSII and PSI probably turns the photosynthetic apparatus into a strong ROS source.

Photosynthesis recovery processed pretty lento, the full recovery of photosynthesis in HT stressed plants required no less than twice the processing time (Li et al., 2009; Wang et al., 2009). However, HL or HH stress resulted in the damage of the photosynthetic apparatus, which triggered the irreversible of photosynthesis (Lichtenthaler and Burkart, 1999). Prior studies recommended that the fast recovery of PSII function was related to either direct reactivation via the reversible conformation change in D1 protein or the relaxation of energy-dependent fluorescence quenching, and more related to CP43 dephosphorylation (Wang H.J. et al., 2003). In this investigation, the recovery of transcription and translation of D1 protein (**Figures 5A**, **6**) was slower than transcription of CP43 (**Figure 5C**) and even fluorescence quenching (**Table 4**). Therefore, fast turnover of the D1 protein is more important to maintain the reaction center activity. As PSI recovery is a tough process affected by several factors, like the protein assembly, the redox state of the electron transporter, and pH gradient across the thylakoid membrane (Shikanai, 2007; Nishiyama and Murata, 2014), more works are needed to explore the precise PSI recovery mechanism.

## Conclusion

We demonstrated that 5 days of sub-high temperature and high light intensity treatment on the basis of our processing material initially caused frustration of mesophyll cells gas diffusion, which increased the intracellular CO_2_ concentration. Whereas the capacity for CO_2_ assimilation was reduced either, so the NADPH utilization was inhibited, which blocked the election transfer of photosynthesis. At the same time, the excess excitation energy caused loss of D1 protein and irreversible inactivation of PSII reaction center. Therefore, the donor and acceptor side of PSI was inhibited respectively. In addition, ROS played an important role in plants. It can be either a response signal of environmental stress or an inhibitor of photosynthesis.

## Author Contributions

Conceived and designed the experiments: TaL and TiL. Performed the experiments: TaL, YL, and ZM. Analyzed the data: TaL, YL, and GZ. Wrote the paper: TaL. Helped revise original paper: TiL, MQ, and ZS.

## Conflict of Interest Statement

The authors declare that the research was conducted in the absence of any commercial or financial relationships that could be construed as a potential conflict of interest.

## Supplementary Material

FIGURE S1 **Phenotypic photographs under different temperature and light intensity**.

FIGURE S2 **Effects of sub-high temperature and high light treatment and recovery on DAB-stained H_2_O_2_ and NBT-stained  levels of tomato leaves; DCFH-DA-stained H_2_O_2_ and DHE-stained  levels in guard cells**.

FIGURE S3 **Effects of sub-high temperature and high light treatment and recovery on soluble protein content (A)**, free proline content **(B)**, relative electrical conductivity, **(C)** and cell damage degree **(D)** of tomato leaves.
