# Supplementary figures and images for: Sub-high Temperature and High Light Intensity Induced Irreversible Inhibition on Photosynthesis System of Tomato Plant (Solanum lycopersicum L.)
Source: Front Plant Sci. 2017 Mar 16;8:365. doi: 10.3389/fpls.2017.00365 (PMC5352666; doi:10.3389/fpls.2017.00365)

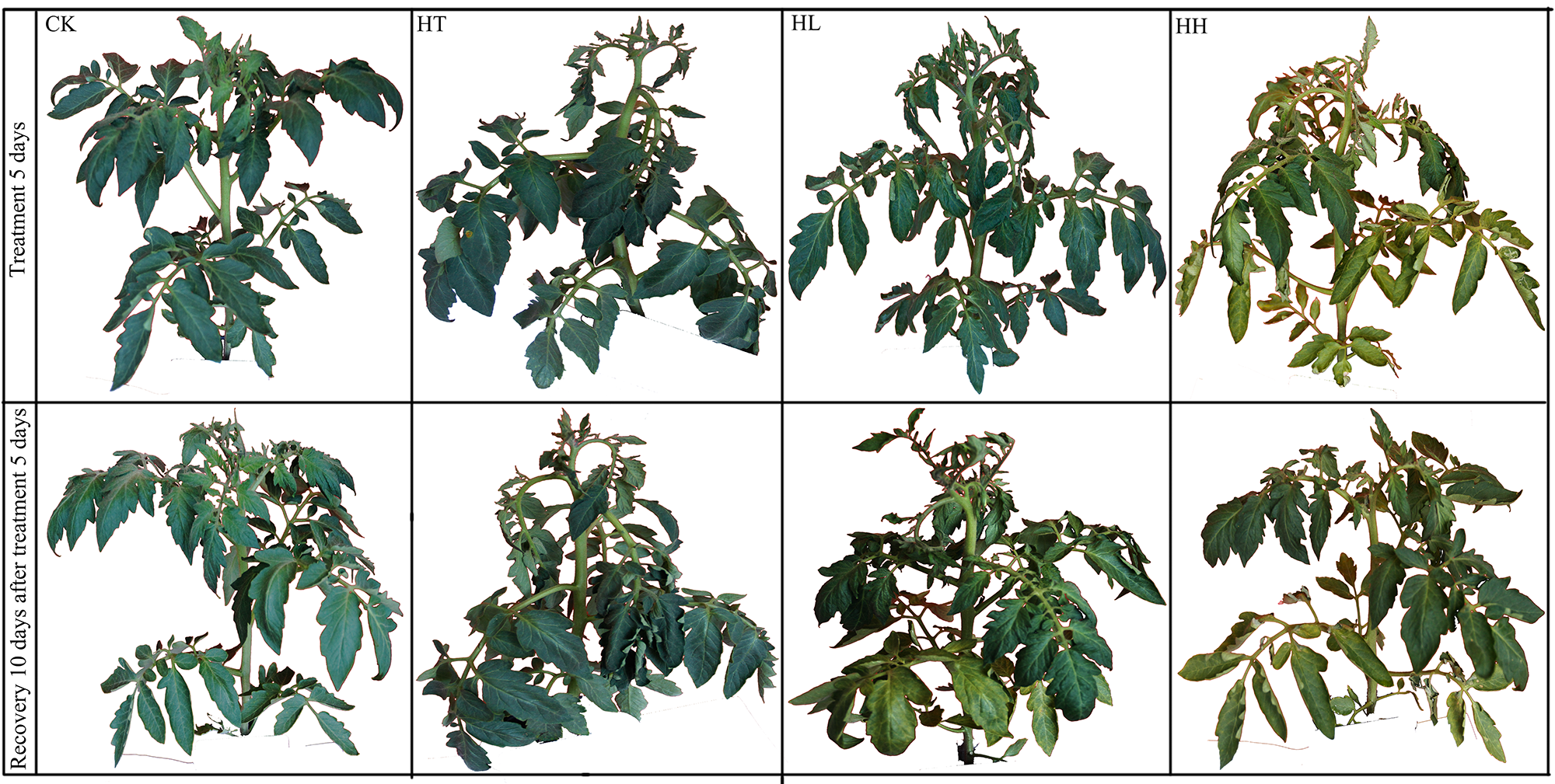

Supplement: FIGURE S1 — Phenotypic photographs under different temperature and light intensity. [file Image_1.TIF]

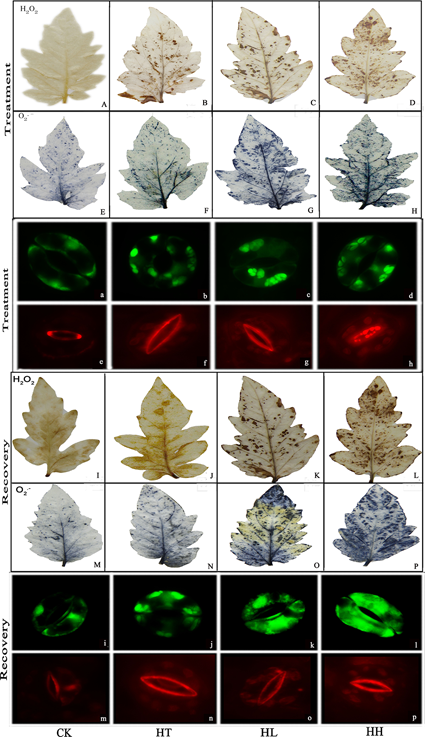

Supplement: FIGURE S2 — Effects of sub-high temperature and high light treatment and recovery on DAB-stained H2O2 and NBT-stained levels of tomato leaves; DCFH-DA-stained H2O2 and DHE-stained levels in guard cells. [file Image_2.TIF]

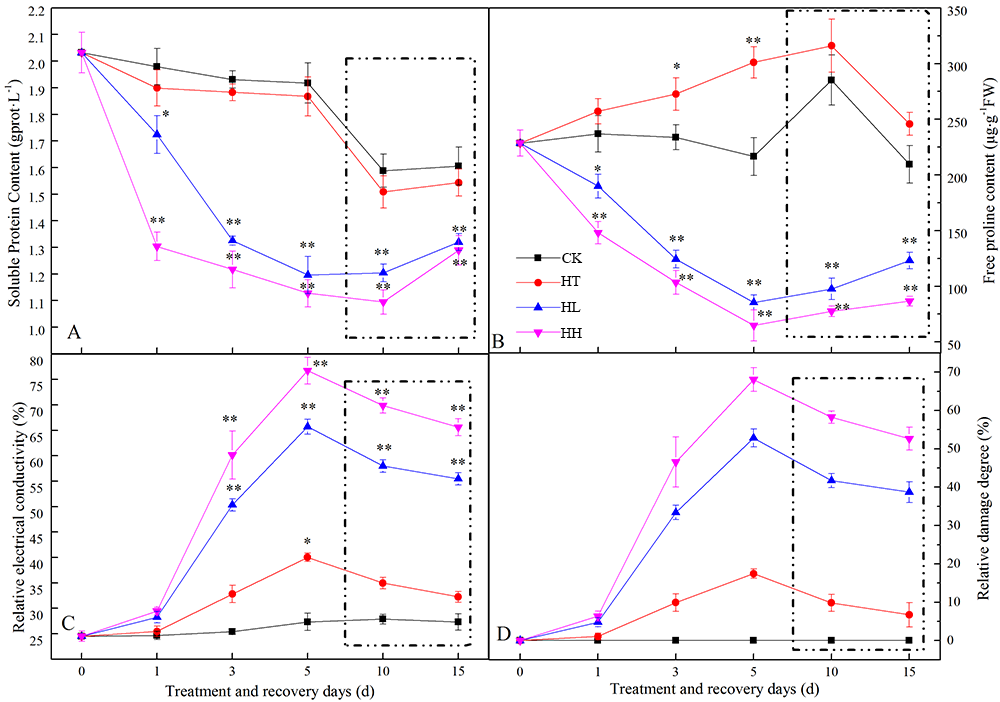

Supplement: FIGURE S3 — Effects of sub-high temperature and high light treatment and recovery on soluble protein content (A), free proline content (B), relative electrical conductivity, (C) and cell damage degree (D) of tomato leaves. [file Image_3.TIF]
