# Supplementary material for: Sub-high Temperature and High Light Intensity Induced Irreversible Inhibition on Photosynthesis System of Tomato Plant (Solanum lycopersicum L.)
Source: Front Plant Sci. 2017 Mar 16;8:365. doi: 10.3389/fpls.2017.00365 (PMC5352666; doi:10.3389/fpls.2017.00365)
Supplement: Supplementary file 4 [file Table_1.DOCX]

**Supplement Table 1 Effects of sub-high temperature and high light intensity treatment and recovery on the content of Chl a, Chlb and lutein of tomato leaves**

| Time(d) | Treatments | Concentraion(mg·g^-1^FW) | | | | Ratio of Chla and Chlb |
| --- | --- | --- | --- | --- | --- | --- |
|  |  | Chlorophyll a | Chlorophyll b | Lutein | Total chlorophyll content |  |
| 0 | Control | 2.37 ±0.15^aA^ | 1.79 ±0.15^aA^ | 0.75 ± 0.14^aA^ | 3.46 ±0.35^aA^ | 2.25 ±0.18^aA^ |
| 1 | HH | 1.77 ±0.24^bB^ | 1.39 ±0.24^bB^ | 1.70 ±0.13^bB^ | 3.16 ±0.17^aA^ | 1.26 ±0.14^bB^ |
|  | HL | 1.94 ±0.24^bAB^ | 1.47 ±0.13^bB^ | 1.04 ±0.13^aA^ | 3.31 ±0.14^aA^ | 1.35 ±0.04^bB^ |
|  | HT | 2.26 ±0.14^aA^ | 1.56 ±0.15^bA^ | 0.94 ±0.33^aA^ | 3.28 ±0.27^aA^ | 1.88 ±0.25^aA^ |
|  | CK | 2.37 ±0.25^aA^ | 1.78 ±0.11^aA^ | 0.84 ±0.15^aA^ | 3.49 ±0.25^aA^ | 2.09 ±0.22^aA^ |
| 3 | HH | 1.67 ±0.29^bB^ | 1.02 ±0.10^cC^ | 2.45 ±0.14^bB^ | 2.69 ±0.30^bB^ | 1.16 ±0.07^bB^ |
|  | HL | 1.88 ±0.15^bB^ | 1.12 ±0.23^bBC^ | 1.34 ±0.13^aA^ | 3.00 ±0.25^bB^ | 1.17 ±0.30^bB^ |
|  | HT | 2.19 ±0.25^aA^ | 1.33 ±0.18^bB^ | 1.11 ±0.27^aA^ | 3.23 ±0.35^bAB^ | 1.46 ±0.14^bA^ |
|  | CK | 2.35 ±0.13^aA^ | 1.63 ±0.13^aA^ | 0.90 ±0.05^aA^ | 3.98 ±0.24^aA^ | 1.99 ±0.17^aA^ |
| 5 | HH | 1.30 ±0.24^cB^ | 0.79 ±0.17^bB^ | 3.23 ±0.13^cB^ | 2.10 ±0.25^cB^ | 1.07 ±0.34^cB^ |
|  | HL | 1.39 ±0.10^cB^ | 0.86 ±0.3^bB^ | 1.51 ±0.37^bA^ | 2.25 ±0.25^cB^ | 1.08 ±0.05^cB^ |
|  | HT | 1.95 ±0.18^bA^ | 0.98 ±0.15^bB^ | 1.23 ±0.03^aA^ | 2.93 ±0.18^bA^ | 1.59 ±0.19^bA^ |
|  | CK | 2.27 ±0.16^aA^ | 1.43 ±0.10^aA^ | 0.89 ±0.14^aA^ | 3.70 ±0.16^aA^ | 1.99 ±0.24^aA^ |
| 10 | HH Recovery | 0.89 ±0.13^bB^ | 0.82 ±0.04^bA^ | 3.50 ±0.14^cC^ | 1.70 ±0.16^cB^ | 1.08 ±0.02^bB^ |
|  | HL Recovery | 1.08 ±0.14^bB^ | 0.87 ±0.15^aA^ | 2.13 ±0.27^bB^ | 1.95 ±0.36^bA^ | 1.18 ±0.19^bB^ |
|  | HT Recovery | 1.50 ±0.24^aA^ | 0.94 ±0.13^aA^ | 1.27 ±0.26^aA^ | 2.44 ±0.14^aA^ | 1.67 ±0.26^aA^ |
|  | CK Recovery | 1.66 ±0.25^aA^ | 1.07 ±0.14^aA^ | 0.93 ±0.24^aA^ | 2.73 ±0.29^aA^ | 1.88 ±0.19^aA^ |
| 15 | HH Recovery | 0.61 ±0.03^bB^ | 0.85 ±0.04^aA^ | 3.36 ±0.16^cB^ | 1.45 ±0.17^bA^ | 0.81 ±0.11^bB^ |
|  | HL Recovery | 0.70 ±0.13^bAB^ | 0.85 ±0.11^aA^ | 2.56 ±0.19^bB^ | 1.55 ±0.19^aA^ | 0.98 ±0.07^bB^ |
|  | HT Recovery | 1.03 ±0.21^aA^ | 0.88 ±0.14^aA^ | 1.59 ±0.25^aA^ | 1.91 ±0.25^aA^ | 1.70 ±0.16^aA^ |
|  | CK Recovery | 1.22 ±0.14^aA^ | 0.88 ±0.17^aA^ | 1.07 ±0.24^aA^ | 2.10 ±0.28^aA^ | 1.79 ±0.27^aA^ |
